# Supplementary material for: Chronic-Antibiotics Induced Gut Microbiota Dysbiosis Rescues Memory Impairment and Reduces β-Amyloid Aggregation in a Preclinical Alzheimer’s Disease Model
Source: Int J Mol Sci. 2022 Jul 26;23(15):8209. doi: 10.3390/ijms23158209 (PMC9331718; doi:10.3390/ijms23158209)
Supplement: Supplementary file 1 [file ijms-23-08209-s001.zip › Table S1.pdf]

Supplementary Table S1. Sequencing summary.

Group	Number of reads	Reads mean per sample	Reads min-max	Length means	Total ASVs counts
		**F0 at GD18-19**			
NoTg-Ctrl	122,771	20,461 ± 9,45	8,873 - 32,242	174.67 ± 4.46	79,954
NoTg-Abx	285,332	71,333 ± 36581	28,540 - 17,980	187 ± 1.63	257,575
3xTg-Ctrl	252,544	31,568 ± 15,374	14,757 - 55,413	174.25 ± 5.42	217,007
3xTg-Abx	255,307	31,913 ± 22,702	3,797 - 69,190	182.875 ± 5.81	226,750
		**F1 PD 30**			
NoTg-Ctrl	303,937	30,393 ± 23484	5876 - 84,662	172.1 ± 6.57	257,206
NoTg-Abx	552,382	55,238 ± 20102	21769 - 82,329	181 ± 6.02	448,468
3xTg-Ctrl	23,2010	23,201 ± 14421	8330 - 56,743	175.3 ± 4.19	196,056
3xTg Abx	34,1234	42654 ± 10298	23419 - 54,525	186.5 ± 1.77	302,518
		**F1 PD 150**			
NoTg-Ctrl	811,364	81,136 ± 44258	5,373 - 121,223	180 ± 7.06	247,542
NoTg-Abx	295,864	29,586 ± 20430	12,100 - 81,839	168.2 ± 7.83	694,128
3xTg-Ctrl	349,078	38,786 ± 27912	3,408 - 78,334	173.44 ± 5.66	293,893
3xTg-Abx	324,969	46,424 ± 29300	15,250- 92,674	182.29 ± 8.94	278,715

**Summary of sequencing after trimming at 200 nt.*

Total of raw read sequences: 3,315,428; Total Mean lengths expressed as bases: 177 ± 8. Identified ASVs: 882, Total ASV counts: 3,499,812.
