# Supplementary material for: Chronic-Antibiotics Induced Gut Microbiota Dysbiosis Rescues Memory Impairment and Reduces β-Amyloid Aggregation in a Preclinical Alzheimer’s Disease Model
Source: Int J Mol Sci. 2022 Jul 26;23(15):8209. doi: 10.3390/ijms23158209 (PMC9331718; doi:10.3390/ijms23158209)
Supplement: Supplementary file 1 [file ijms-23-08209-s001.zip › Table S2.pdf]

Supplementary Table S2. Relative abundance of fecal samples from NoTg and 3xTg mice.

F0 at GD18-19	NoTg Ctrl	NoTg Abx	*p*- value
Proteobacteria	20.44 ± 0.43	75.53 ± 0.42	0.074
Bacteroidetes	42.99 ± 0.28	14.14 ± 0.31	0.092
Firmicutes	29.32 ± 0.22	4.81 ± 0.10	0.065
Actinobacteria	0.01 ± 0.00	0.27 ± 0.03	0.058
Others	1.03 ± 0.06	0.75 ±.0.00	0.714
	**3xTg Ctrl**	**3xTg Abx**	***p*- value**
Proteobacteria	4.17 ± 0.02	87.15 ± 0.21	<0.0001
Bacteroidetes	68.84 ± 0.11	0.04 ± 0.00	<0.0001
Firmicutes	21.46 ± 0.09	0.22 ± 0.00	<0.0001
Actinobacteria	0.01 ± 0.00	0.08 ± 0.00	0.3501
Others	1.36 ± 0.04	4.17 ± 0.02	0.580
**F1at PD30**	**NoTg Ctrl**	**NoTg Abx**	***p*- value**
Proteobacteria	2.35 ± 0.02	90.10 ± 0.05	<0.0001
Bacteroidetes	60.67 ± 0.13	0.00 ± 0.00	<0.0001
Firmicutes	19.87 ± 0.09	0.00 ± 0.00	<0.0001
Actinobacteria	0.07 ± 0.00	0.00 ± 0.00	0.1667
Others	17.04 ± 0.06	0.90 ± 0.05	0.853
**F1at PD30**	**3xTg Ctrl**	**3xTg Abx**	***p*- value**
Proteobacteria	5.01 ± 0.02	93.07 ± 1.66	<0.0001
Bacteroidetes	58.93 ± 0.13	0.00 ± 0.00	<0.0001
Firmicutes	19.87 ± 0.09	0.04 ± 0.08	<0.0001
Actinobacteria	0.07 ± 0.00	0.08 ± 0.23	0.035
Others	9.75 ± 0.03	6.55 ± 0.02	0.163
**F1at PD150**	**NoTg Ctrl**	**NoTg Abx**	***p*- value**
Proteobacteria	2.27 ± 0.03	86.03 ± 0.14	<0.0001
Bacteroidetes	45.55 ± 0.21	0.45 ± 0.01	<0.0001
Firmicutes	31.78 ± 0.19	3.91 ± 0.11	0.0003
Actinobacteria	0.73 ± 0.01	0.2 ± 0.00	0.7214
Others	19.66 ± 0.08	9.41 ± 0.05	0.510
**F1at PD150**	**3xTg Ctrl**	**3xTg Abx**	***p*- value**
Proteobacteria	3.51 ± 0.03	65.86 ± 0.43	0.040
Bacteroidetes	48.90 ± 0.21	19.72 ± 0.33	0.086
Firmicutes	30.29 ± 0.04	3.92 ± 0.06	0.001
Actinobacteria	2.30 ± 0.04	0.16 ± 0.00	0.185
Others	15.00 ± 0.05	10.33 ± 0.03	0.600

Data shows the Mean ± standard deviation as indicated. The *p*-values were calculated using the Mann-Whitney U test. $p < 0.05$ are considered statistically significant.
