# Supplementary material for: Chronic-Antibiotics Induced Gut Microbiota Dysbiosis Rescues Memory Impairment and Reduces β-Amyloid Aggregation in a Preclinical Alzheimer’s Disease Model
Source: Int J Mol Sci. 2022 Jul 26;23(15):8209. doi: 10.3390/ijms23158209 (PMC9331718; doi:10.3390/ijms23158209)
Supplement: Supplementary file 1 [file ijms-23-08209-s001.zip › Table S3.pdf]

Supplementary Table S3. Alpha-diversity indexes of fecal samples from NoTg and 3xTg mice.

Index	NoTg-Ctrl	NoTg-Abx	*p*- value
**F0 at GD18-19**			
[a]Chao1 index	103.40 ± 49.81	15.50 ± 6.61	0.014
[b]Shannon index	0.36 ± 0.36	0.91 ± 0.16	0.014
[b]Simpson index	0.92 ± 0.03	0.39 ± 0.08	0.016
	**3xTg-Ctrl**	**3xTg-Abx**	
[a]Chao1 index	125.63 ± 68.41	12.63 ± 8.14	< 0.001
[b]Shannon index	3.85 ± 0.26	0.89 ± 0.40	< 0.001
[b]Simpson index	0.96 ± 0.01	0.40 ± 0.15	< 0.001
**F1 at PD 30**			
	**NoTg Ctrl**	**NoTg Abx**	
[a]Chao1 index	1055.31 ± 219.18	12.5 ± 2.37	< 0.001
[b]Shannon index	4.30 ± 0.22	1.06 ± 0.17	< 0.001
[b]Simpson index	0.96 ± 0.01	0.46 ± 0.08	< 0.001
	**3xTg Ctrl**	**3xTg Abx**	
[a]Chao1 index	98.90 ± 46.29	13.63 ± 1.65	< 0.001
[b]Shannon index	3.51 ± 0.69	0.95 ± 0.13	< 0.001
[b]Simpson index	0.92 ± 0.09	0.40 ± 0.06	< 0.001
**F1 at PD 150**			
	**NoTg Ctrl**	**NoTg Abx**	
[a]Chao1 index	1055 ± 219.18	1018.65 ± 344.21	< 0.001
[b]Shannon index	4.30 ± 0.22	3.98 ± 0.54	< 0.001
[b]Simpson index	0.96 ± 0.01	0.94 ± 0.05	< 0.001
	**3xTg Ctrl**	**3xTg Abx**	
[a]Chao1 index	100.22 ± 53.05	31.14 ± 32.86	< 0.001
[b]Shannon index	3.47 ± 0.52	1.75 ± 1.24	0.042
[b]Simpson index	0.93 ± 0.04	0.59 ± 0.25	0.012

Data shows the Mean ± standard deviation as indicated (see Figs. x and x). The *p*-values were calculated using Mann-Whitney U testcomparing data of Ctrl vs. Abx in NoTg and 3xTg mouse in mothers (F0) and offspring (F1). $p < 0.05$ are considered statistically significant.
