# Supplementary material for: Chronic-Antibiotics Induced Gut Microbiota Dysbiosis Rescues Memory Impairment and Reduces β-Amyloid Aggregation in a Preclinical Alzheimer’s Disease Model
Source: Int J Mol Sci. 2022 Jul 26;23(15):8209. doi: 10.3390/ijms23158209 (PMC9331718; doi:10.3390/ijms23158209)
Supplement: Supplementary file 1 [file ijms-23-08209-s001.zip › Table S4.pdf]

Supplementary Table 4. Linear discriminant analysis (LDA) scores were computed for bacterial differentially abundant (order, family, and genus).

Bacteria	LDA	*p*- value	*q*-value
**F0 3xTg-Ctrl**			
f_S24-7	5.5629	0.0005	0.0051
g_ Lactobacillus	3.3776	0.0043	0.0282
g_Odoribacter	3.8558	0.0011	0.0084
g_Clostridium	3.9208	0.0011	0.0084
g_Bacteroides	4.9983	0.0003	0.0034
o_Clostridiales	4.9646	0.0003	0.0034
f__Lachnospiraceae	4.7685	0.0003	0.0034
g__Paraprevotella	4.3644	0.0003	0.0034
f__Rikenellaceae	4.2801	0.0003	0.0034
f__Paraprevotellaceae_g__Prevotella	4.7645	0.0003	0.0034
f__Ruminococcaceae	4.1134	0.0003	0.0034
f__Prevotellaceae_g__Prevotella	4.7275	0.0003	0.0034
g__Oscillospira	4.0017	0.0037	0.0261
g__Dehalobacterium	2.6281	0.0272	0.1668
o__Bacteroidales	4.4661	0.0011	0.0084
**F0 3xTg-Abx at PD30**			
f__Enterobacteriaceae	3.6351	0.0272	0.6256
g__Klebsiella	4.9506	0.0006	0.0322
g__Sutterella	4.6674	0.0100	0.3066
g__Enterobacter	5.8677	0.0007	0.0322
**F1 3xTg-Ctrl at PD30**			
f S24 7	5.5646	0.0002	0.0046
f__Rikenellaceae_g__AF12	3.4837	0.0110	0.0532
g__Lactobacillus	4.9443	0.0002	0.0046
g__Parabacteroides	3.3236	0.0245	0.0980
g__Streptococcus	3.6290	0.0245	0.0980
g__Bacteroides	5.0075	0.0002	0.0046
o__Clostridiales	4.7971	0.0009	0.0111
g_Prevotella	4.5567	0.0017	0.0111
f__Ruminococcaceae_g__Ruminococcus	3.3869	0.0017	0.0111
f__Lachnospiraceae_g___Ruminococcus	3.6134	0.0017	0.0111
f__Desulfovibrionaceae	3.3822	0.0017	0.0111
f__Rikenellaceae	4.4614	0.0002	0.0046
f__Lachnospiraceae	4.5938	0.0009	0.0110
f__Ruminococcaceae	3.9310	0.0046	0.0248
g__Odoribacter	3.8225	0.0046	0.0248
g__Prevotella	4.5770	0.0006	0.0110
f__Clostridiaceae_g__CandidatusArthromitus	3.8482	0.0110	0.0532
f__Erysipelotrichaceae	3.1710	0.0017	0.0111
g__Oscillospira	4.1941	0.0017	0.0111
o__Bacteroidales	3.3253	0.0017	0.0111
g__Flexispira	3.5329	0.0245	0.0980
f__Helicobacteraceae	3.3771	0.0046	0.2948
g__Coprobacillus	2.8083	0.0245	0.0980
f__Rikenellaceae_g__AF12	3.4837	0.0110	0.0532

**F1 3xTg-Abx at PD30**			
g__*Mycoplasma*	3.1694	0.0128	0.2940
g__*Klebsiella*	5.0103	0.0029	0.0889
g__*Sutterella*	4.8105	0.0005	0.0230
g__*Enterobacter*	5.8821	0.0002	0.0184
**F1 NoTg-Ctrl at PD30**			
o__Bacteroidales_f__S24_7	5.5400	0.0000	0.0000
g__*Lactobacillus*	3.8395	0.0001	0.0010
g__*Parabacteroides*	3.5146	0.0305	0.1476
g__*Bacteroides*	4.7280	0.0000	0.0000
o__Clostridiales	5.0571	0.0000	0.0000
g___*Prevotella*	4.2382	0.0051	0.0335
f_Lachnospiraceae_g__Ruminococcus	3.6263	0.0006	0.0050
f__Ruminococcaceae_g___Ruminococcus	3.6501	0.0130	0.0747
f__Rikenellaceae	5.0976	0.0000	0.0000
f__Lachnospiraceae	4.3621	0.0000	0.0000
g__Edwardsiella	4.1715	0.0130	0.0747
f__Ruminococcaceae	4.1587	0.0001	0.0010
g__*Prevotella*	4.6131	0.0000	0.0000
c__Alphaproteobacteria	3.4730	0.0051	0.0335
f__Clostridiaceae_g__CandidatusArthromitus	3.8461	0.0019	0.0145
f__Erysipelotrichaceae	2.9173	0.0305	0.1476
g__*Oscillospira*	4.2932	0.0000	0.0000
g__*Dehalobacterium*	2.542	0.0305	1.0000
o__Bacteroidales	4.0835	0.0006	0.0050
g__*Flexispira*	2.9978	0.0305	0.0145
**F1 3xTg-Ctrl at PD150**			
o__Bacteroidales_f__S24_7	5.5248	0.0468	0.2443
f__Rikenellaceae_g__AF12	3.4295	0.0150	0.1533
g__*Lactobacillus*	4.4110	0.0276	0.2116
g__*Odoribacter*	3.6578	0.0234	0.2032
g__*Dorea*	4.3169	0.0106	0.1393
g__*Clostridium*	3.8163	0.0023	0.0705
g__*Allobaculum*	4.7245	0.0364	0.2232
o__Clostridiales	5.0991	0.0090	0.1393
f__Lachnospiraceae	4.5570	0.0023	0.0703
g__Paraprevotella	3.5331	0.0243	0.2032
f__Desulfovibrionaceae	3.9334	0.0034	0.0782
f__Rikenellaceae	4.4931	0.0015	0.0705
g__Ruminococcus	3.4270	0.0106	0.1393
f__Ruminococcaceae	3.9809	0.0361	0.2232
g__*Bifidobacterium*	4.3100	0.0309	0.2186
g__*Prevotella*	4.8369	0.0468	0.2443
g__*Oscillospira*	4.0087	0.0124	0.1426
f__Helicobacteraceae	3.9994	0.0478	0.2443
**F1 3xTg-Abx at PD150**			
g__*Mycoplasma*	3.5450	0.0123	0.2829
f__Enterobacteriaceae	3.9462	0.0412	0.7580
g__*Klebsiella*	4.7940	0.0089	0.2760
g__*Sutterella*	4.7879	0.0035	0.2760
g__*Enterobacter*	5.7191	0.0090	0.2760

**F1 NoTg-Ctrl at PD150**			
o__Bacteroidales_f__S24_7	5.5223	0.0000	0.0000
g__*Coprococcus*	2.9091	0.0305	0.1753
f__Rikenellaceae_g__AF12	3.3451	0.0359	0.1942
g__*Lactobacillus*	4.7896	0.0000	0.0000
g__*Clostridium*	3.8841	0.0305	0.1753
g__*Bacteroides*	3.8112	0.0016	0.0184
o__Clostridiales	5.1807	0.0003	0.0046
g___*Prevotella*	4.5118	0.0001	0.0023
g__*Ruminococcus*	3.4279	0.0450	0.2300
g___*Ruminococcus*_	3.7308	0.0118	0.0904
f__Rikenellaceae	4.5612	0.0003	0.0046
f__Lachnospiraceae	4.7433	0.0011	0.0144
f__Ruminococcaceae	3.9085	0.0037	0.0377
g__*Odoribacter*	3.8747	0.0081	0.6774
g__*Prevotella*	4.5482	0.0000	0.0000
f__Erysipelotrichaceae	2.8093	0.0305	0.1753
g__*Oscillospira*	3.9837	0.0041	0.0372
g__*Mycoplasma*	2.6120	0.0130	0.0920
**F1 NoTg-Abx at PD150**			
f__Enterobacteriaceae	4.4381	0.0000	0.0000
g__*Klebsiella*	4.7965	0.0000	0.0000
g__*Sutterella*	5.0282	0.0001	0.0030
g__*Enterobacter*	5.8133	0.0001	0.0023

The threshold on the logarithmic LDA score was set to 2.5. p and q values < 0.05 were considered statistically significant. *p-value was adjusted with Benjamini-Hochberg method and generated FDR value (q-value). The name of each taxon level appears in abbreviation before its taxon. “f”, family; “g”, genus. “LDA” Linear discriminant analysis.
