# Supplementary material for: Chronic-Antibiotics Induced Gut Microbiota Dysbiosis Rescues Memory Impairment and Reduces β-Amyloid Aggregation in a Preclinical Alzheimer’s Disease Model
Source: Int J Mol Sci. 2022 Jul 26;23(15):8209. doi: 10.3390/ijms23158209 (PMC9331718; doi:10.3390/ijms23158209)
Supplement: Supplementary file 1 [file ijms-23-08209-s001.zip › Table S5.pdf]

Table S5. Correlation between bacterial diversity and histological measures and behavioral test in 3xTg at PD150 mice.

Parameter	*p*-value	r	R^2
**Total- and 1-42 Aβ in the dorsal hippocampus**			
Aβ1-42 area in *subiculum*	0.0179	0.7871	0.6195
Aβ1-42 area in CA1	0.0726	0.6108	0.3730
Aβ1-42 area in CA3	0.0086	0.8428	0.7103
Total Aβ area in *subiculum*	0.0012	0.9305	0.8658
Total Aβ area in CA1	0.0030	0.8985	0.8073
Total Aβ area in CA3	0.0007	0.9441	0.8914
**Novel object localization**			
NOL_Training_Preference	0.0633	-0.3982	0.1585
NOL_Retention_Preference	0.0113	-0.5648	0.3190

Pearson correlation coefficients were considered significant with *p* values <0.05.
