# Supplementary material for: Chronic-Antibiotics Induced Gut Microbiota Dysbiosis Rescues Memory Impairment and Reduces β-Amyloid Aggregation in a Preclinical Alzheimer’s Disease Model
Source: Int J Mol Sci. 2022 Jul 26;23(15):8209. doi: 10.3390/ijms23158209 (PMC9331718; doi:10.3390/ijms23158209)
Supplement: Supplementary file 1 [file ijms-23-08209-s001.zip › Table S6.pdf]

Supplementary Table 6. Spearman Correlation *p* values for immunohistochemistry data in F1 3xTg mice at PD150.

Bacteria	Total βA area in *subiculum*	Total βA area in CA3	Total βA area in CA1	βA1-42 area in *subiculum*	βA1-42 area in CA3	βA1-42 area in CA1
f_Lachnospiraceae	0.037	0.162	0.096	0.027	0.027	0.470
f_Rikenellaceae_g_AF12	0.037	0.162	0.096	0.027	0.027	0.470
g_*Clostridium*	0.078	0.188	0.062	0.019	0.019	0.280
f_Desulfovibrionaceae	0.068	0.162	0.213	0.028	0.010	0.483
g_*Oscillospira*	0.186	0.302	0.524	0.091	0.048	0.742
o_Bacteroidales	0.444	0.406	0.608	0.129	0.048	0.483
g_*Prevotella*	0.220	0.170	0.194	0.016	0.016	0.170
g_*Streptococcus*	0.162	0.526	0.122	0.039	0.225	0.685
g_*Paraprevotella*	0.261	0.577	0.078	0.027	0.192	0.429
g_*Parabacteroides*	0.429	0.651	0.280	0.049	0.162	0.513
g_*Bacteroides*	0.247	0.091	0.170	0.050	0.194	0.108
g_*Ruminococcus*	0.027	0.122	0.122	0.319	0.122	0.912
g_*Dorea*	0.027	0.122	0.526	0.319	0.122	0.912
g_*Anaeroplasma*	0.027	0.122	0.526	0.319	0.122	0.912
g_*Turicibacter*	0.039	0.078	0.577	0.406	0.078	0.912
g_*Lactobacillus*	0.050	0.050	0.444	0.091	0.050	0.601
g_*Klebsiella*	0.096	0.019	0.062	0.019	0.019	0.188
g-*Enterobacter*	0.140	0.068	0.162	0.038	0.006	0.335
f_F16.g	0.188	0.027	0.062	0.162	0.188	0.019
g_*Mycoplasma*	0.039	0.027	0.526	0.027	0.027	0.027
f_Enterobacteriaceae.	0.019	0.008	0.188	0.008	0.008	0.062
g_*Allobaculum*	0.742	0.406	0.261	0.162	0.039	0.078
g_*Sutterella*	0.040	0.035	0.011	0.003	0.076	0.050

Note: immunohistochemistry data of area occupied for total- and 1-42 βA in the *subiculum*, CA1, and CA3. *p-value* < 0.05 were considered significant.
