# Supplementary material for: Chronic-Antibiotics Induced Gut Microbiota Dysbiosis Rescues Memory Impairment and Reduces β-Amyloid Aggregation in a Preclinical Alzheimer’s Disease Model
Source: Int J Mol Sci. 2022 Jul 26;23(15):8209. doi: 10.3390/ijms23158209 (PMC9331718; doi:10.3390/ijms23158209)
Supplement: Supplementary file 1 [file ijms-23-08209-s001.zip › Table S7.pdf]

Table 7. Spearman Correlation p values for the 22 genera identified in gut microbiota in behavior performance and motor activity.

Bacteria	Exploration time_Nov	MA Distance	MA Resting Time	Retention Preference	Training Preference
g_*Bifidobacterium*	0.220	0.268	0.003	0.0001	0.052
f_Coriobacteriaceae	0.104	0.717	0.381	0.107	0.013
g_*Adlercreutzia*	0.397	0.597	0.735	0.404	0.010
o_Bacteroidales	0.586	0.314	0.246	0.045	0.789
g_*Bacteroides*	0.436	0.049	0.054	0.040	0.514
g_*Paraprevotella*	0.611	0.958	0.295	0.011	0.071
g_*Prevotella*	0.778	0.279	0.135	0.027	0.354
g_*Rikenella*	0.995	0.531	0.878	0.042	0.575
g_*Mucispirillum*	0.328	0.383	0.625	0.094	0.035
g_*Lactobacillus*	0.139	0.276	0.097	0.010	0.046
g_*Streptococcus*	0.050	0.578	0.667	0.055	0.001
g_*Clostridium*	0.192	0.937	0.321	0.046	0.367
g_*Dahalobacterium*	0.589	0.348	0.089	0.028	0.921
g_*Dorea*	0.040	1	0.104	0.000	0.055
g_*Lachnospira*	0.494	0.429	0.746	0.042	0.199
f_Ruminococcaceae	0.859	0.042	0.061	0.193	0.407
g_*Allobaculum*	0.152	0.209	0.003	0.003	0.005
c_Alphaproteobacteria	0.886	0.223	0.026	0.128	0.202
f_Desulfovibrionaceae	0.097	0.188	0.007	0.000	0.122
g_*Edwadsiella*	0.049	0.467	0.442	0.334	0.997
g_*Anaeroplasma*	0.163	0.491	0.237	0.005	0.767
g_*Akkermansia*	0.847	0.592	0.266	0.031	0.060

Note: Nov: novel-object localization, MA: Motor activity represents significant differences ($p < 0.05$)
