# Supplementary figures and images for: Chronic-Antibiotics Induced Gut Microbiota Dysbiosis Rescues Memory Impairment and Reduces β-Amyloid Aggregation in a Preclinical Alzheimer’s Disease Model
Source: Int J Mol Sci. 2022 Jul 26;23(15):8209. doi: 10.3390/ijms23158209 (PMC9331718; doi:10.3390/ijms23158209)

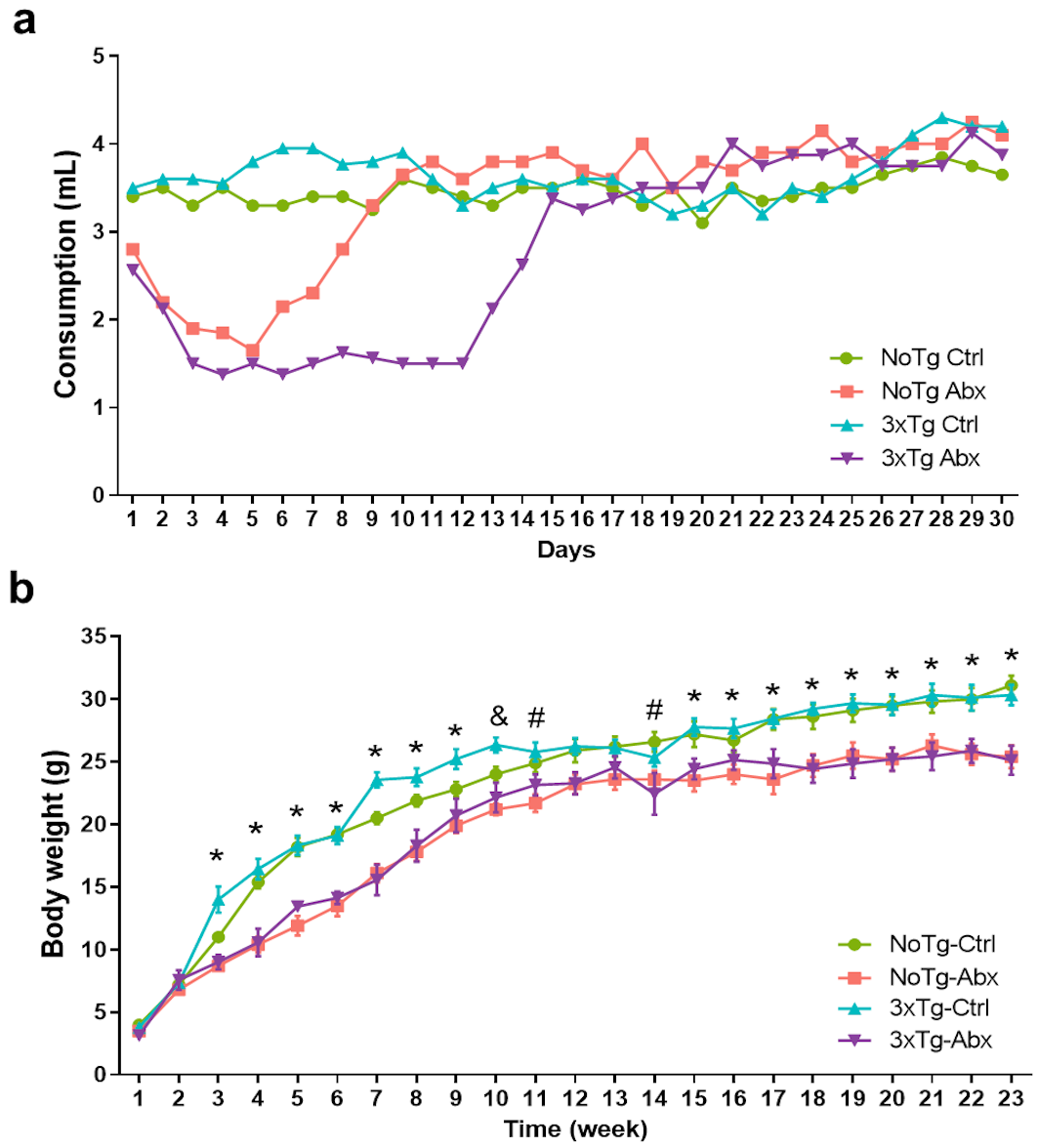

Supplement: Supplementary file 1 [file ijms-23-08209-s001.zip › SF 1.tif]

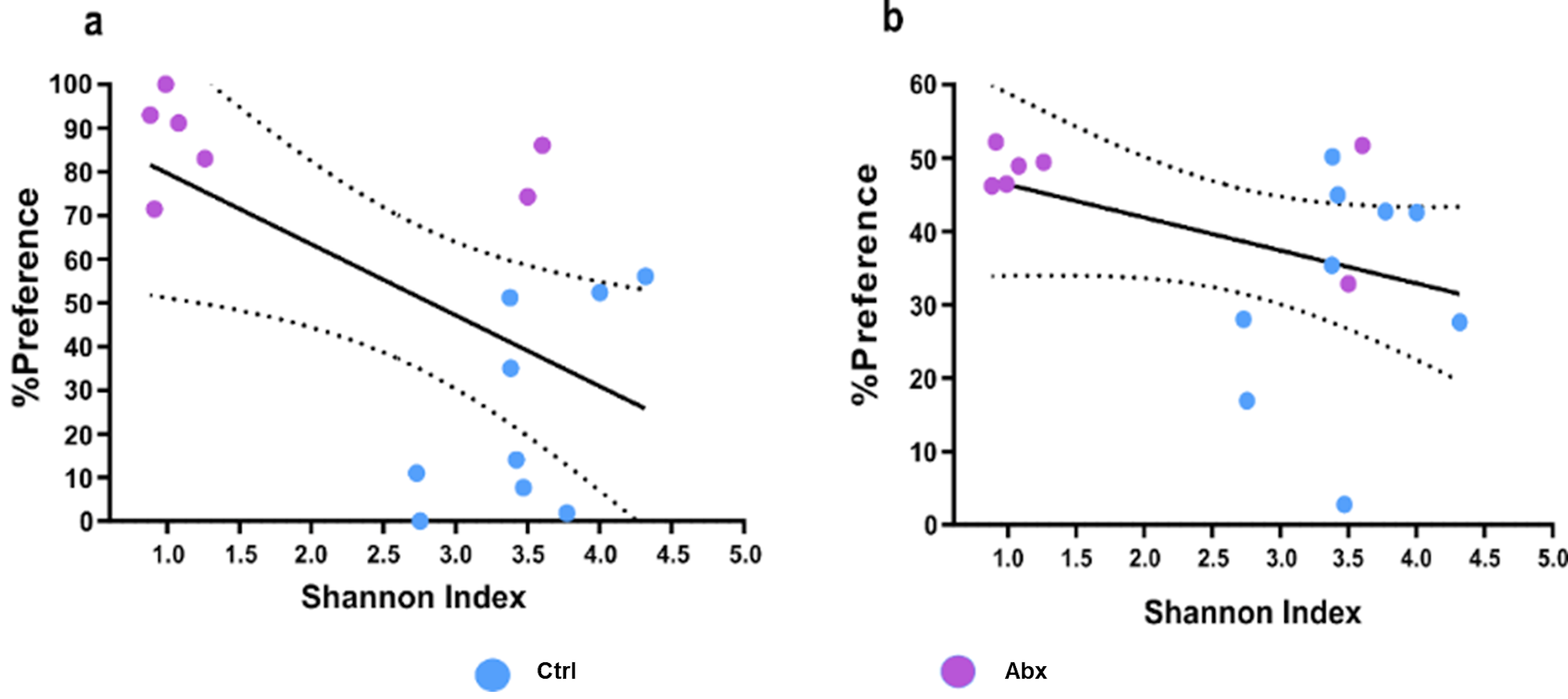

Supplement: Supplementary file 1 [file ijms-23-08209-s001.zip › SF 10.tif]

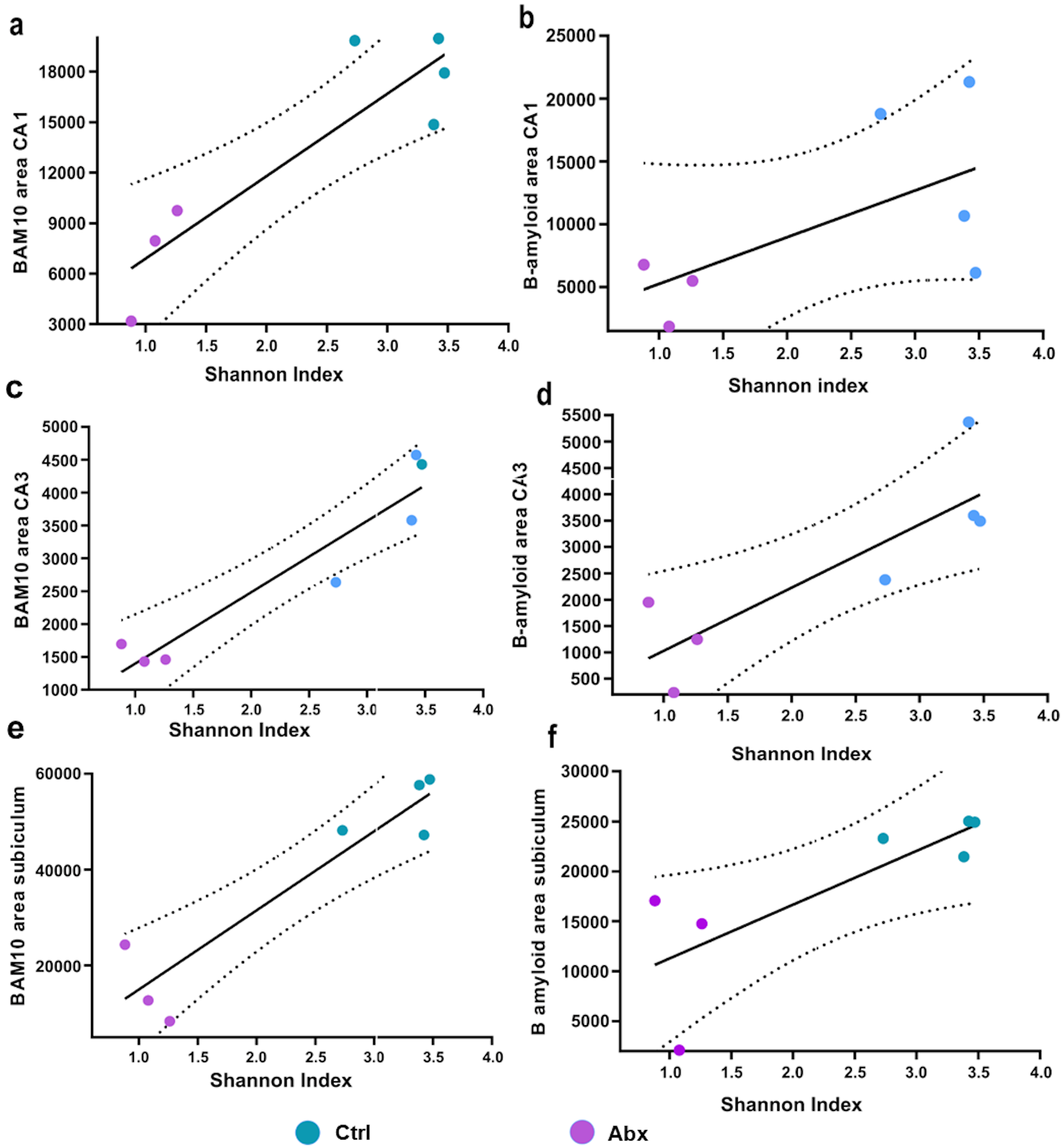

Supplement: Supplementary file 1 [file ijms-23-08209-s001.zip › SF 11.tif]

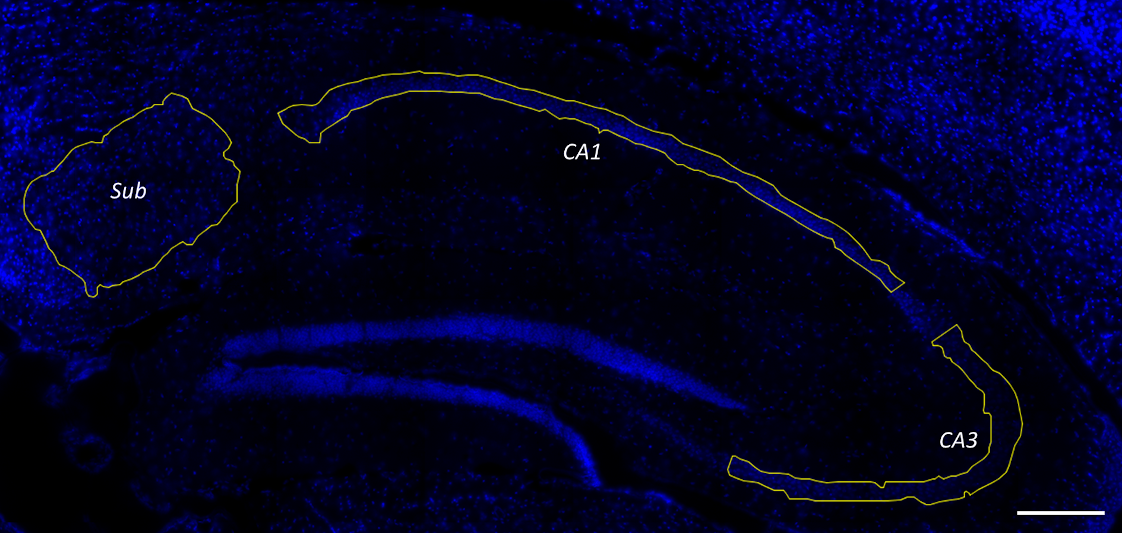

Supplement: Supplementary file 1 [file ijms-23-08209-s001.zip › SF 2.tif]

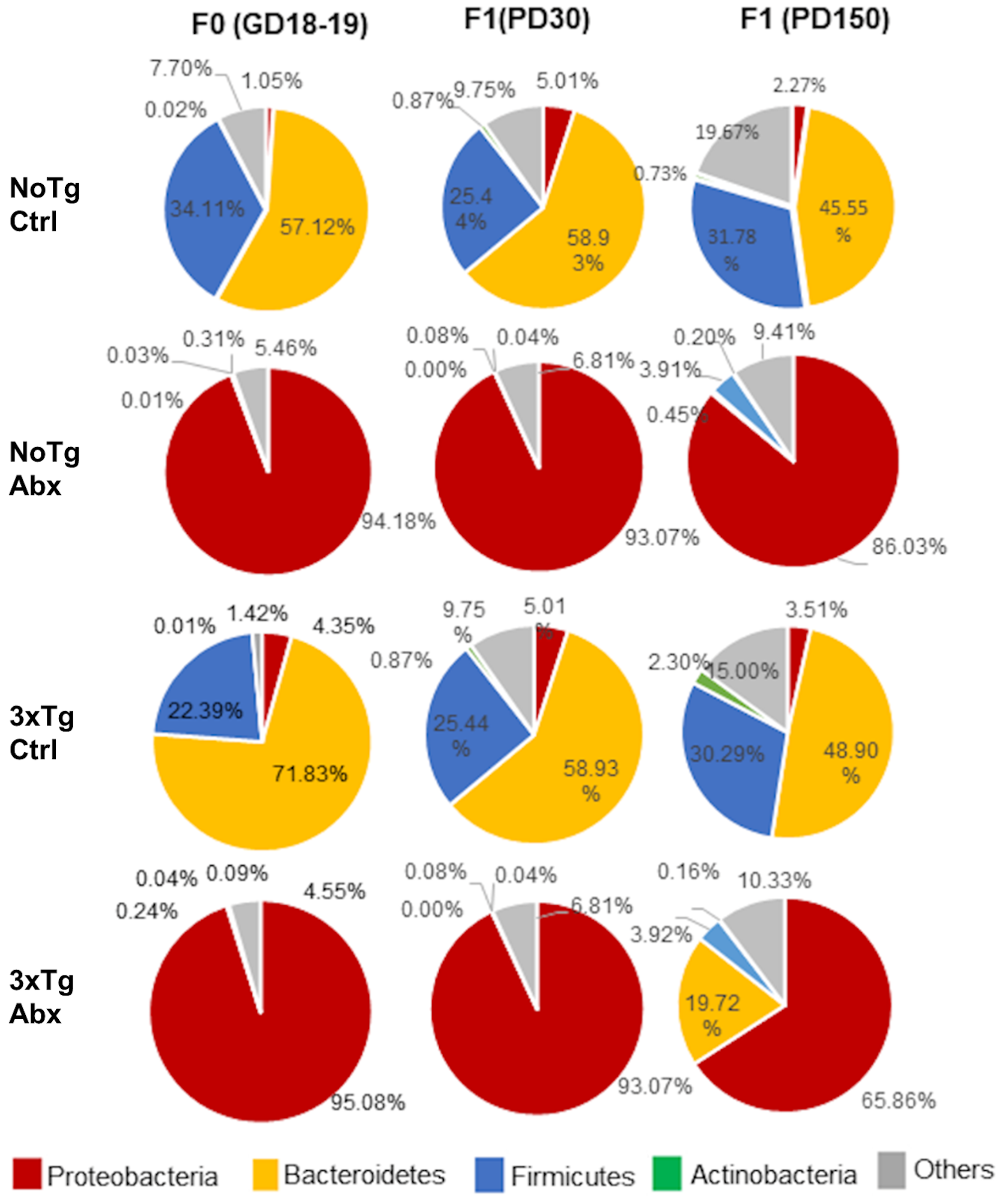

Supplement: Supplementary file 1 [file ijms-23-08209-s001.zip › SF 3.tif]

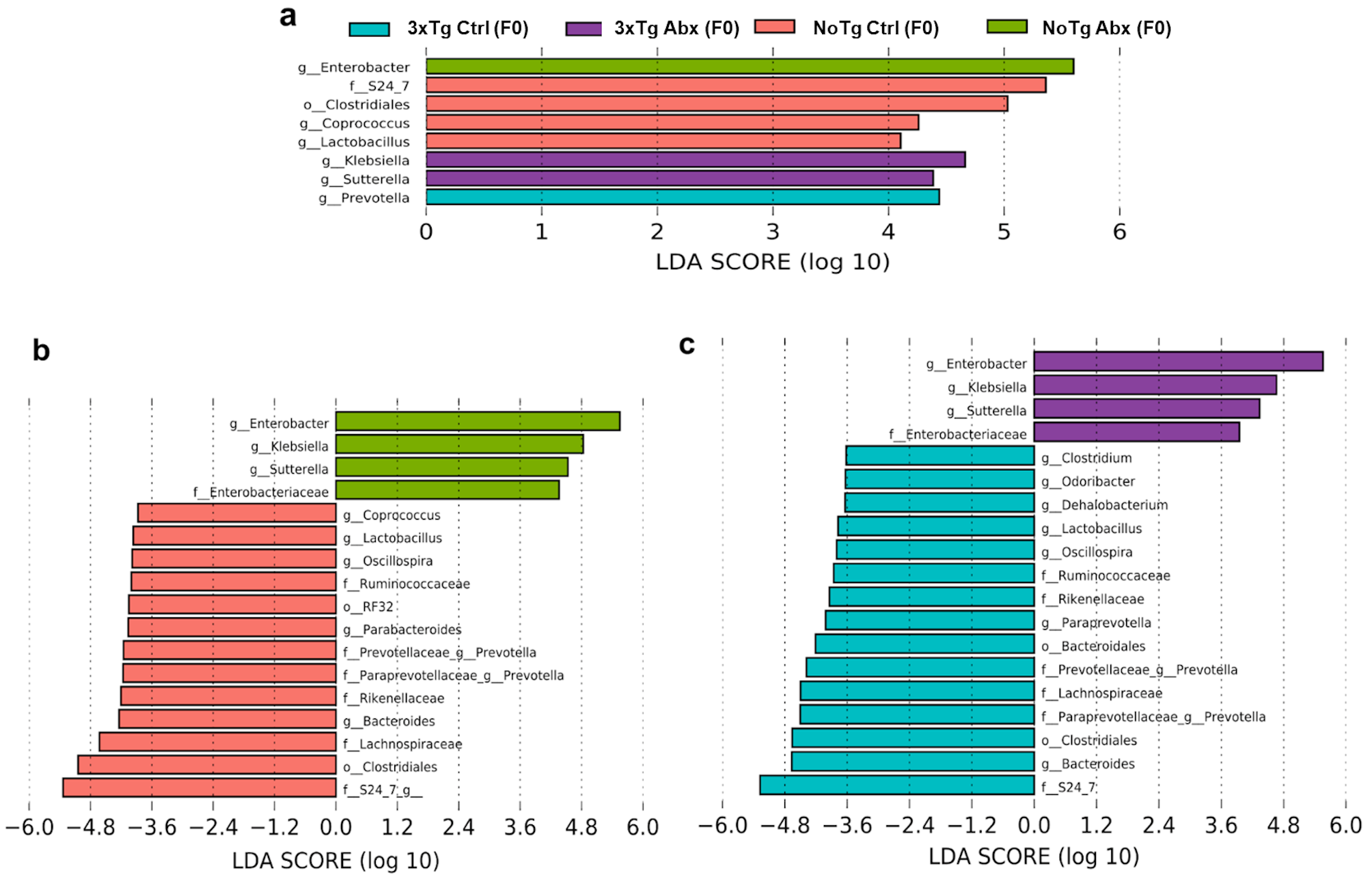

Supplement: Supplementary file 1 [file ijms-23-08209-s001.zip › SF 4.tif]

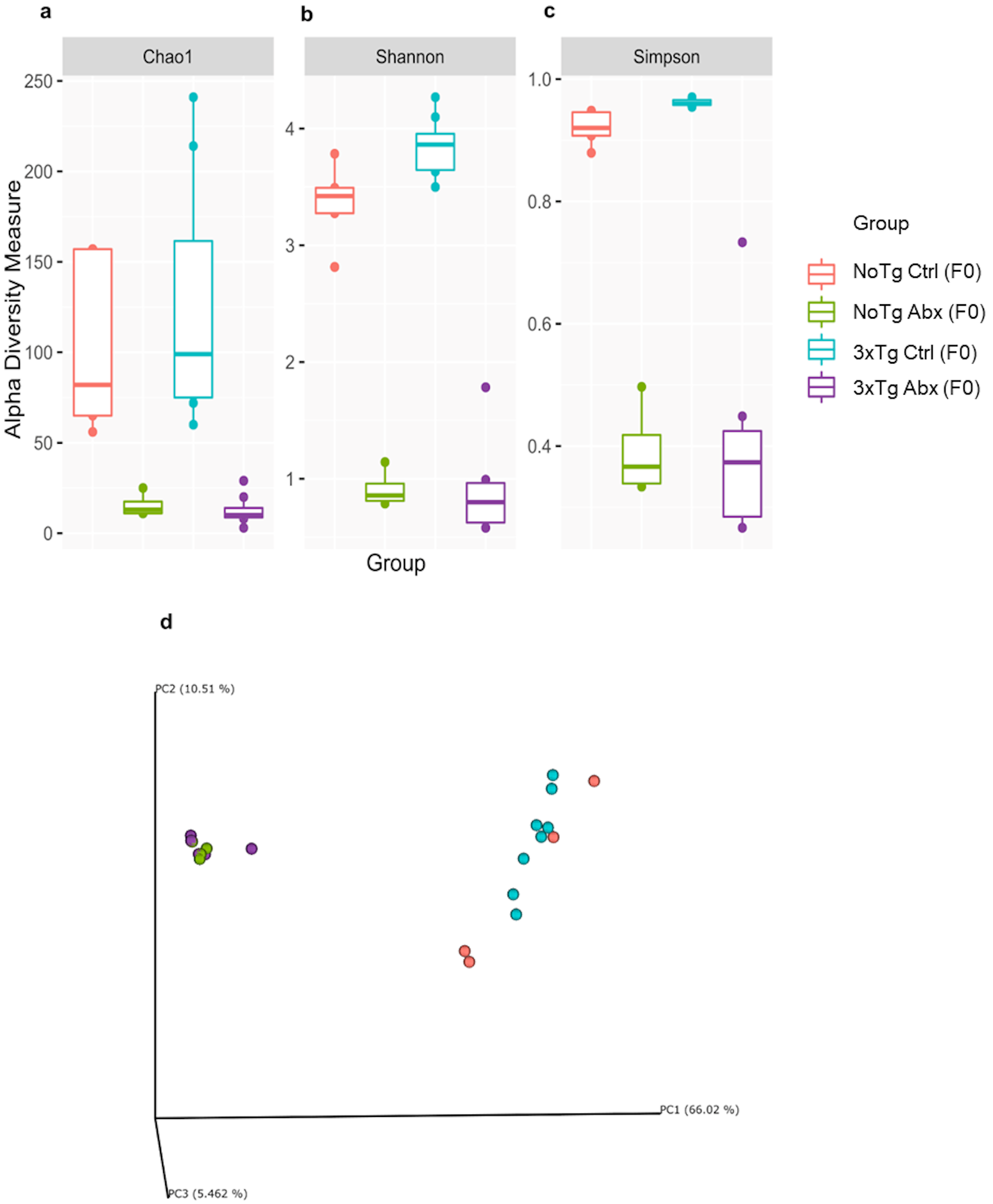

Supplement: Supplementary file 1 [file ijms-23-08209-s001.zip › SF 5.tif]

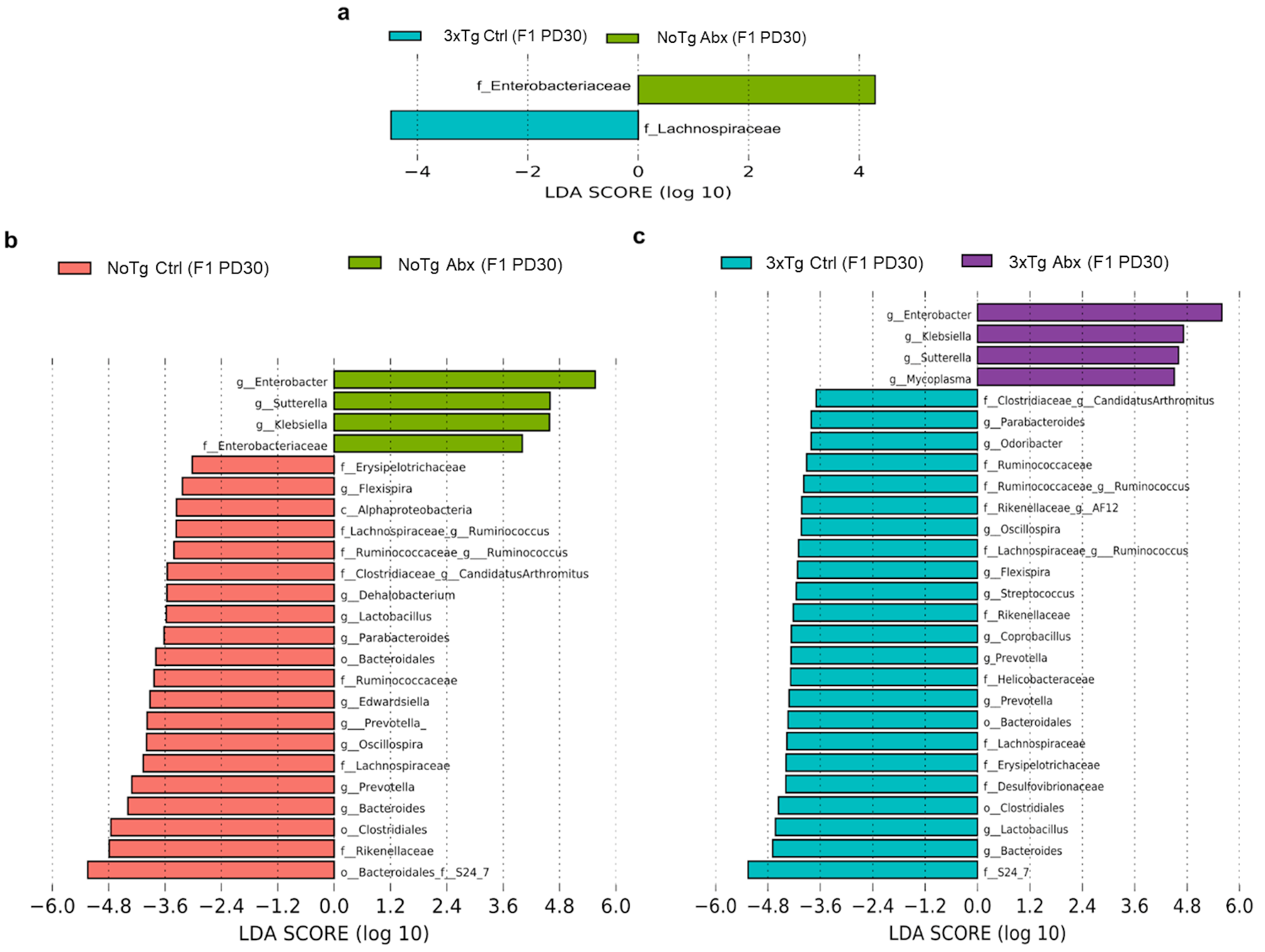

Supplement: Supplementary file 1 [file ijms-23-08209-s001.zip › SF 6.tif]

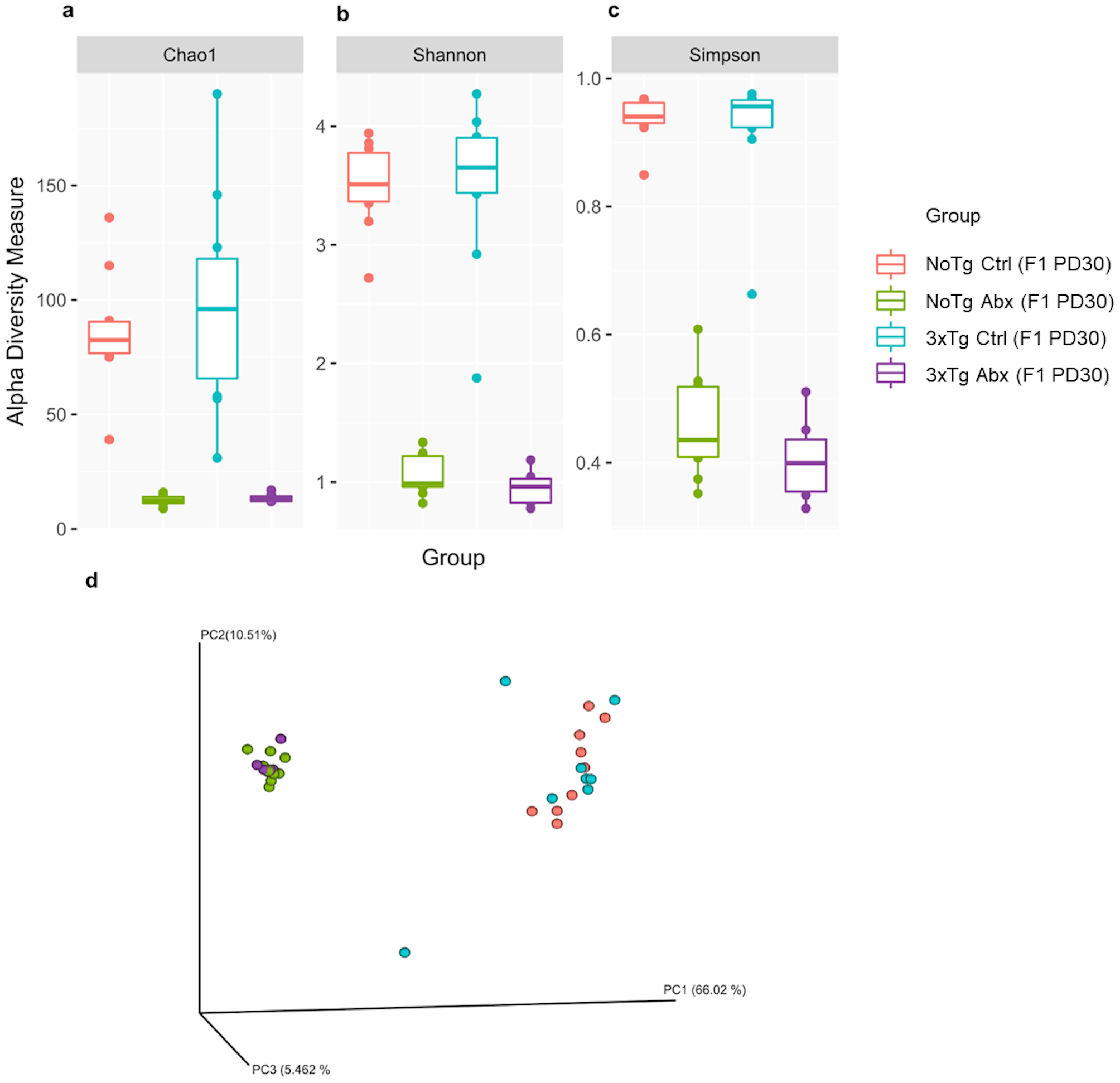

Supplement: Supplementary file 1 [file ijms-23-08209-s001.zip › SF 7.tif]

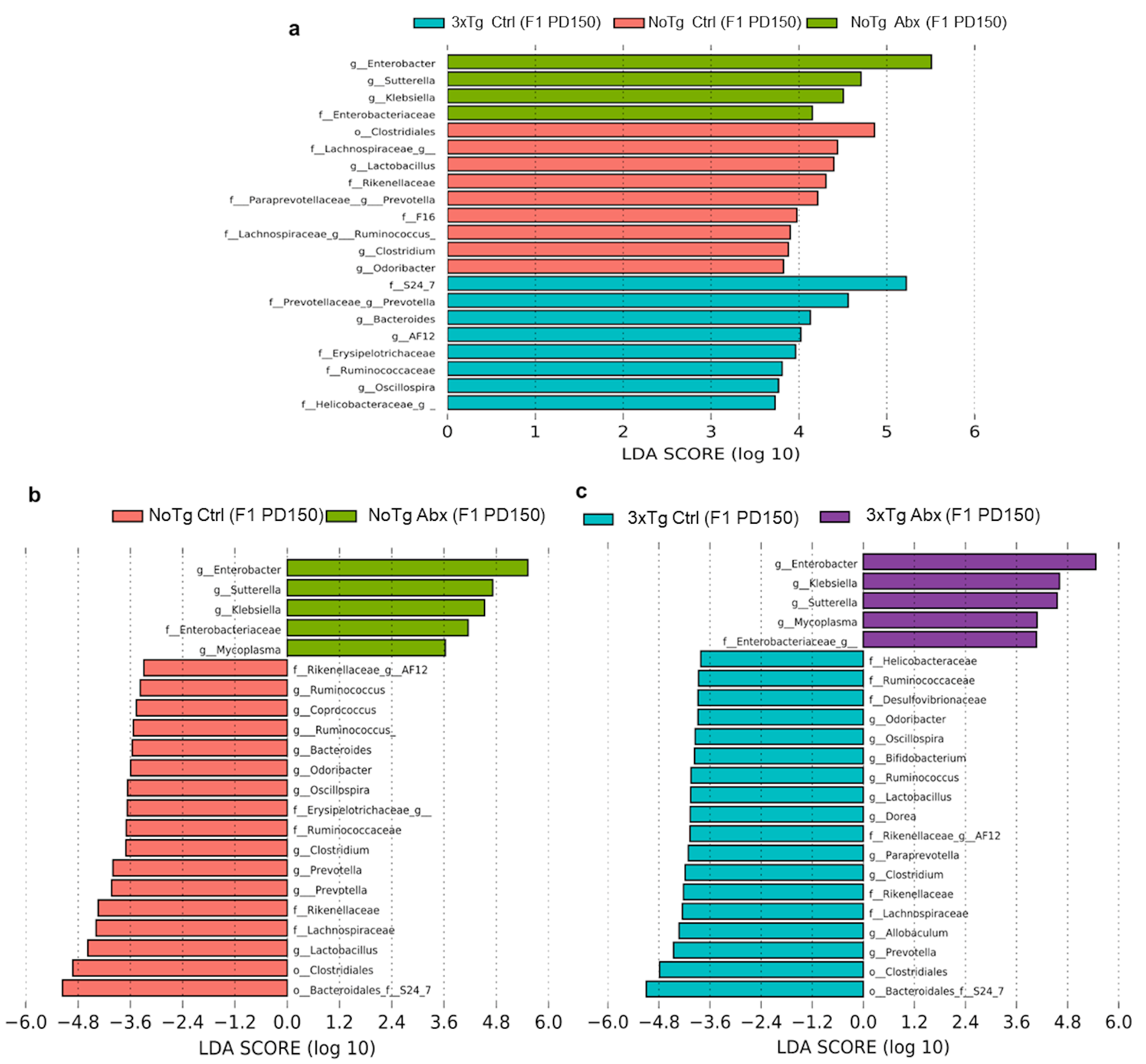

Supplement: Supplementary file 1 [file ijms-23-08209-s001.zip › SF 8.tif]

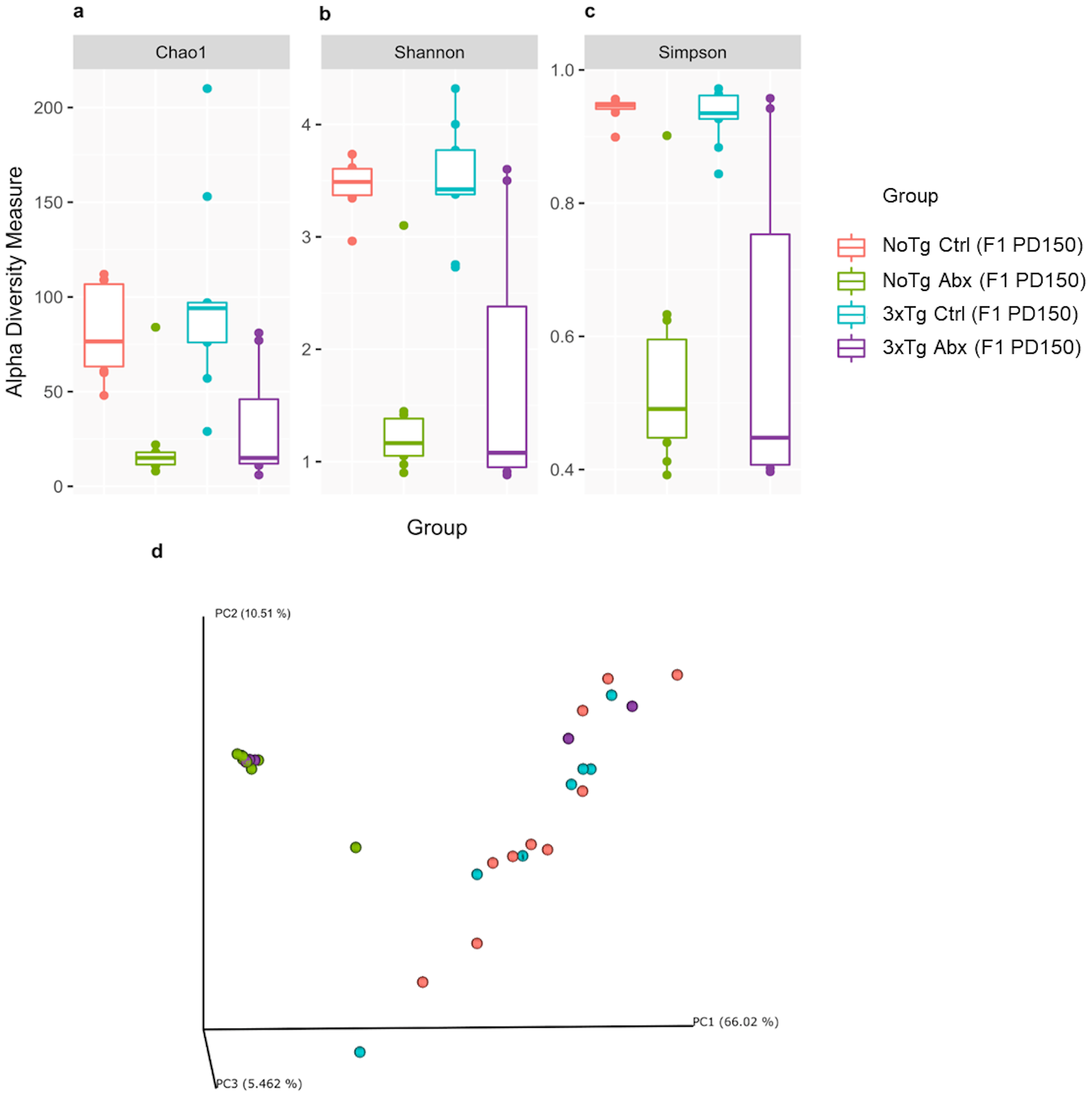

Supplement: Supplementary file 1 [file ijms-23-08209-s001.zip › SF 9.tif]
